# A Treatise of Electrolysis

**Published:** 1886-11

**Authors:** 


**A Treatise of Electrolysis** and its application to Therapeutical and Surgical Treatment in Disease. By ROBERT AMORY, A M., M. D., Professor of Physiology in Bowdoin College. New York: Wm. Wood & Co. 1886.

This volume presents to the profession, in a condensed form, that knowledge of electricity necessary for the intelligent employment of this force in medicine. It defines its limits of therapeutical application, so that the physician may know how to apply electricity to the human structure in a rational way, and to withhold its application in these cases of diseased tissues which are not amenable to its favorable action. The book can be read with advantage by every one who uses elec tricity in his practice—and in this day who does not?

